# Synthesis, crystal structure and Hirshfeld surface analysis of 2-azido-*N*-(2,6-di­methyl­phen­yl)acetamide

**DOI:** 10.1107/S2056989025004530

**Published:** 2025-06-17

**Authors:** Abderrazzak El Moutaouakil Ala Allah, Benson M. Kariuki, Ahlam I. Al-Sulami, Maram T. Basha, Basmah H. Allehyani, Abdulsalam Alsubari, Joel T. Mague, Youssef Ramli

**Affiliations:** a Laboratory of Medicinal Chemistry Drug Sciences Research Center Faculty of Medicine and Pharmacy Mohammed V University in Rabat Morocco; b School of Chemistry, Cardiff University, Main Building, Park Place, Cardiff, CF10 3AT, United Kingdom; c University of Jeddah, Jeddah 21589, Saudi Arabia; d Laboratory of Medicinal Chemistry, Faculty of Clinical Pharmacy, 21 September University, Yemen; e Department of Chemistry, Tulane University, New Orleans, LA, 70118, USA; Venezuelan Institute of Scientific Research, Venezuela

**Keywords:** crystal structure, azide, phenyl­acetamide, hydrogen bond, C—H⋯π(ring) inter­action

## Abstract

The asymmetric unit of the title compound, C_10_H_12_N_4_O, consists of two independent mol­ecules differing in the rotational orientation of the 2-azido­acetamido group. In the crystal, inspection of the contacts of the methyl groups shows an intra­molecular H⋯O distance of 2.47 Å in one mol­ecule and inter­molecular H⋯N distances of 2.75 Å in both independent mol­ecules. Both are definitely van der Waals contacts with the latter quite short as the H⋯O distance is 0.39 Å less than the sum of the respective van der Waals radii. A Hirshfeld surface analysis indicates that the H⋯H contacts make the largest contribution. In the absence of any specific C—H⋯N hydrogen bonds, the significant contribution of N⋯H/H⋯N contacts (24.7%) might seem surprising, but with the azide group projecting away from the rest of the mol­ecule, there is considerable opportunity for such contacts to occur.

## Abstract 2

The asymmetric unit of the title compound, C_10_H_12_N_4_O, consists of two independent mol­ecules differing in the rotational orientation of the 2-azido­acetamido group.

## 1. Chemical context

**Figure 1:**
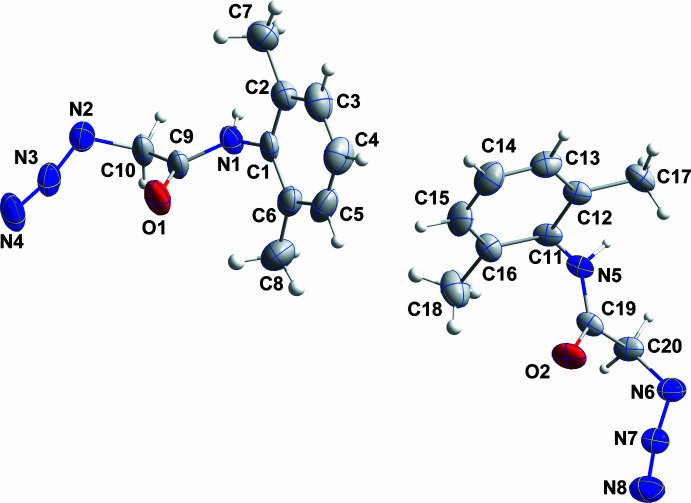
The asymmetric unit with 50% probability ellipsoids for non-hydrogen atoms and 5% probability ellipsoids for hydrogen atoms.

Amides play an essential role in the structure of numerous natural products, agrochemicals, peptides, polymers, proteins, biologically active compounds, and functional materials (Humphrey & Chamberlin, 1997 ▸). The amide bond is among the most remarkable functional groups in nature due to its strong polarity, high stability, and conformational versatility (Wieland & Bodanszky, 2012 ▸). Furthermore, amides participate in a wide range of functional group transformations and organic reactions, enabling the synthesis of nitriles, carbonyl compounds, esters, amino acids, azides, amines, hydro­carbons, and pharmaceutical compounds. (Lectka, 2001 ▸). Among the compounds derived from *N*-aryl­acetamides under the action of sodium azide (Scriven & Turnbull, 1988 ▸; Missioui *et al.*, 2022*a* ▸), azides stand out for their valuable applications in medicinal chemistry and mol­ecular biology (Khandelwal *et al.*, 2024 ▸). Increasingly studied in organic synthesis, they play a key role as inter­mediates in the preparation of heterocycles such as triazolines and triazoles, typically formed through 1,3-dipolar cyclo­addition reactions (Tron *et al.*, 2008 ▸). Herein we report the synthesis and spectroscopic characterization of the new azide derived from *N*-aryl­acetamide **3**. A colorless plate-like specimen of the title compound (Fig. 1 ▸) was used for the X-ray crystallographic analysis. A Hirshfeld surface analysis was performed to analyze the inter­molecular inter­actions.
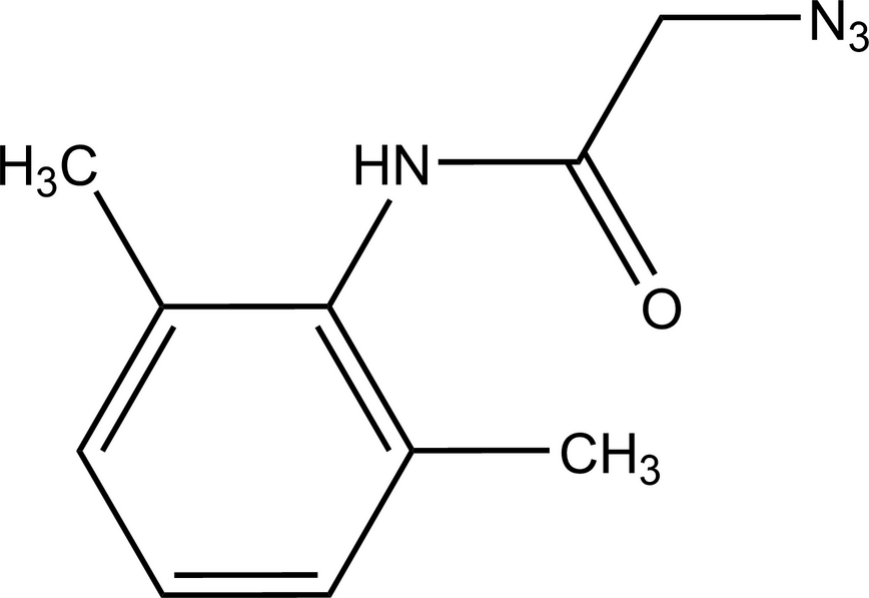


## 2. Structural commentary

The asymmetric unit consists of two independent mol­ecules differing in the rotational orientation of the 2-azido­acetamido group. Thus, the O1—C9—N1—C1 and the C9—C10—N2—N3 torsion angles in the first mol­ecule are −6.3 (9) and −86.3 (7)°, respectively, while in the second mol­ecule, the O2—C19—N5—C11 and the C19—C20—N6—N7 torsion angles are 6.8 (8) and 86.6 (7)°, respectively. The sums of angles about N1 and N5 are both 360° within experimental error, indicating involvement of their lone pairs in N→C π bonding. This occurs primarily with the carbonyl carbon atom as expected with the N1—C9 and N1—C1 distances being 1.351 (7) and 1.430 (7) Å, respectively, and the N5—C19 and the N5—C11 distances at 1.350 (6) and 1.433 (6) Å, respectively. The dihedral angle between the mean plane of the C1—C6 phenyl ring and that defined by C1, N1, C9 and O1 is 60.6 (4)° while the corresponding angle in the second mol­ecule is 61.4 (3)°. These angles are considerably larger than the corresponding ones in the most closely related mol­ecules (*vide infra*) and are likely due to steric considerations resulting from the presence of the two methyl groups *ortho* to the acetamido group. Inspection of the contacts of the C7 and C8 methyl groups shows an intra­molecular distance H8*B*⋯O1 of 2.47 Å and an inter­molecular distance H7*B*⋯N4 (at −*x*, −*y* + 1, −*z*) of 2.75 Å. Both are definitely van der Waals contacts but with the former having an H⋯O distance 0.39 Å less than the sum of the respective van der Waals radii, one might consider it a C—H⋯O hydrogen bond. However, the C—H⋯O angle is less than 120° so it is best considered a very short van der Waals contact. The contacts are oriented such that a diminution of the above-mentioned dihedral angle would decrease both these distances, which would be unfavorable. For the second mol­ecule, a similar situation obtains for the C17 and C18 methyl groups with an intra­molecular H18*A*⋯O2 contact of 2.86 Å and an inter­molecular H17*B*⋯N8 (at −*x* + 1, −*y* + 1, −*z* + 1) contact of 2.75 Å, both about the sum of the relevant van der Waals radii. Again, a diminution of the dihedral angle here would shorten these contacts.

## 3. Supra­molecular features

**Figure 2:**
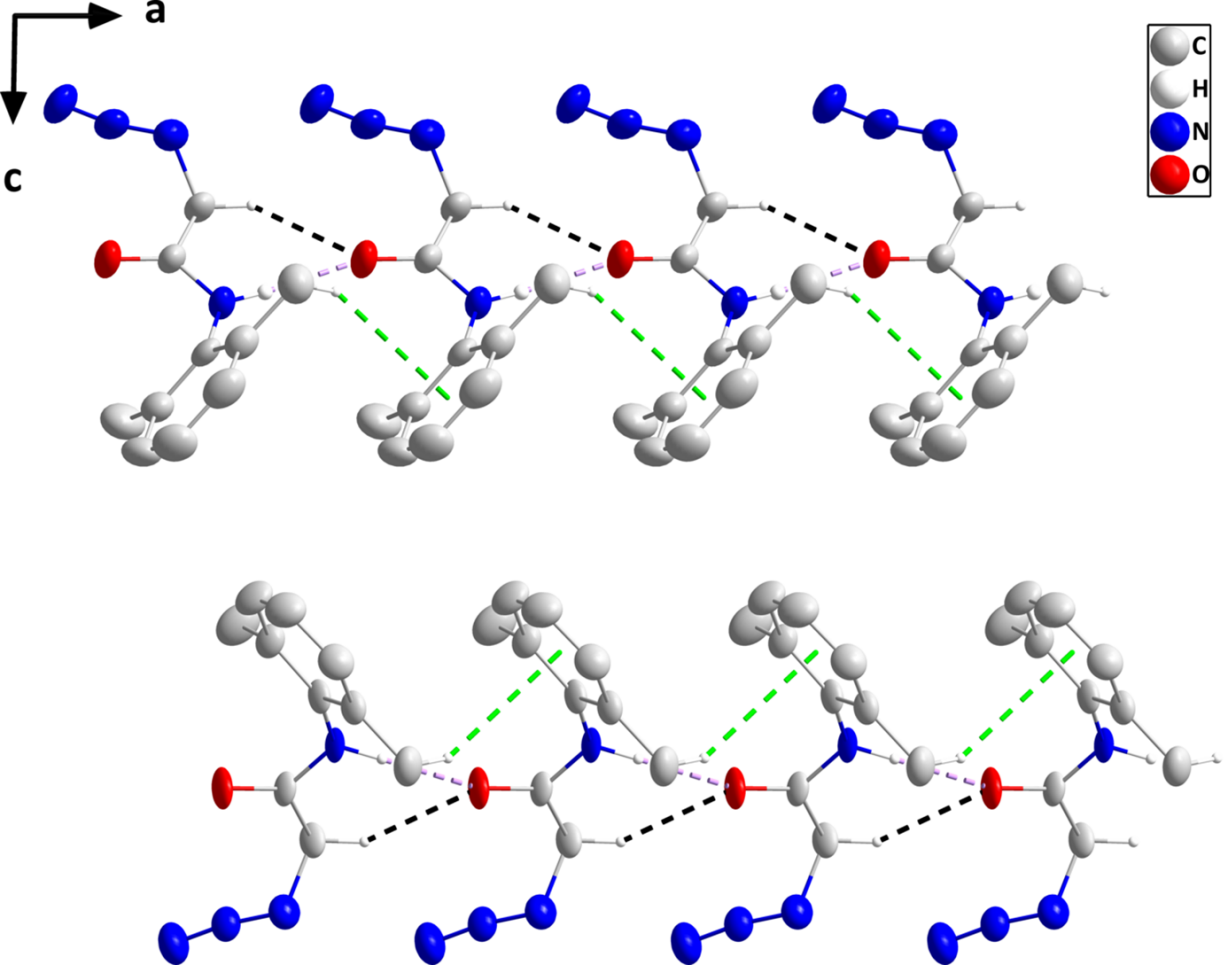
Portions of the two independent chains viewed along the *b*-axis direction with N—H⋯O and C—H⋯O hydrogen bonds depicted, respectively, by violet and black dashed lines. The C—H⋯π(ring) inter­actions are depicted by green dashed lines and hydrogen atoms not involved in these inter­actions are omitted for clarity.

**Figure 3:**
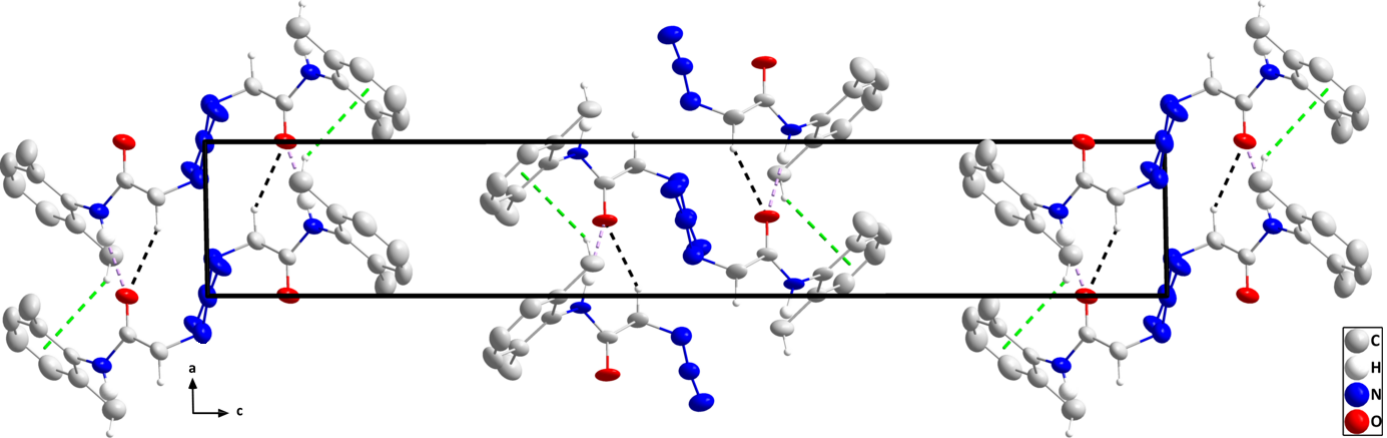
Packing viewed along the *b*-axis direction with N—H⋯O and C—H⋯O hydrogen bonds depicted, respectively, by violet and black dashed lines. The C—H⋯π(ring) inter­actions are depicted by green dashed lines and hydrogen atoms not involved in these inter­actions are omitted for clarity.

In the crystal, chains of the mol­ecule containing N1 and extending along the *a*-axis direction are formed by N1—H1⋯O1 hydrogen bonds and reinforced by C10—H10*A*⋯O1 hydrogen bonds and C7—H7*C*⋯*Cg*1 inter­actions (Table 1 ▸). Analogous chains of the mol­ecule containing N5 are formed by N5—H5*A*⋯O2 and C20—H20*B*⋯O2 hydrogen bonds plus C17—H17*A*⋯*Cg*2 inter­actions (Table 1 ▸ and Fig. 2 ▸). The chains pack with largely normal van der Waals contacts (Fig. 3 ▸).

## 4. Database survey

**Figure 4:**
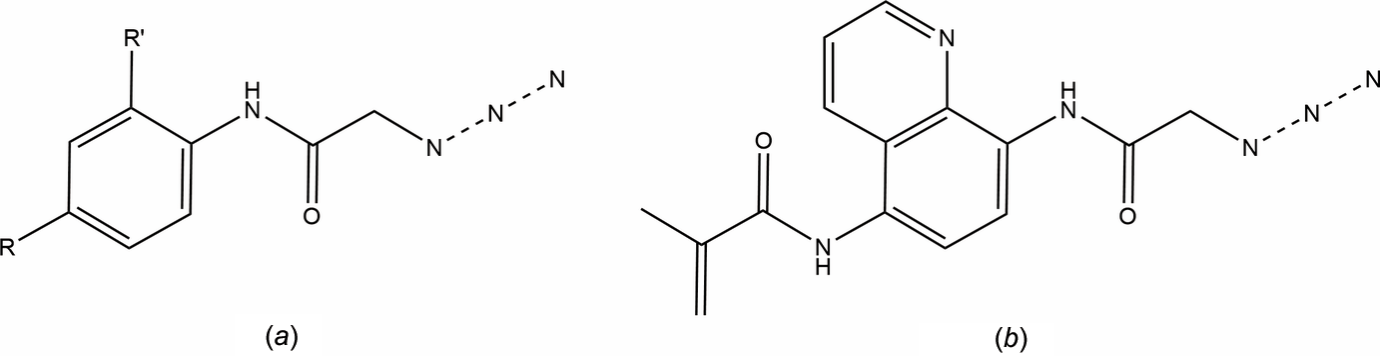
The search fragment used for the database survey (*a*) and LETTIR (*b*).

A search of the Cambridge Structural Database (CSD, updated to January 2025; Groom *et al.*, 2016 ▸) with the search fragment shown in Fig. 4 ▸*a* (*R* = *R*′ = nothing) generated 24 hits of which 10 were similar to the title mol­ecule. The remainder were triazole derivatives. The similar mol­ecules have *R* = *R*′ = H (ASEDIO; Guerrab *et al.*, 2021 ▸) and *R*′ = H, *R* = (2,3,4,6-tetra-*O*-acetyl-α-d-galacto­pyran­oside) (BEBPIJ; Cecioni *et al.*, 2012 ▸), Me (BEKRES; Missioui *et al.*, 2022*a* ▸), F (BEKRIW; Missioui *et al.*, 2022*b* ▸), *R* = (C≡CH) (DAPYOM; Madhusudhanan *et al.*, 2021 ▸. DAPYOM01; Raju *et al.*, 2023 ▸), NO_2_ (QAGNOF; Missioui *et al.*, 2020 ▸) and OMe (TARHIH; Missioui *et al.*, 2022*d* ▸). Of the last two, one has *R* = Cl and *R*′ = 2-chloro­benzoyl (VIFVOX; Cortes-Maya *et al.*, 2012 ▸) and the other is shown in Fig. 4 ▸*b* (LETTIR; Guirado-Moreno *et al.*, 2023 ▸). As in the present structure, the asymmetric units of ASEDIO, BEKRIW, DAPYOM, DAPYOM01, LETTIR and VIFVOX consist of two independent mol­ecules (*Z*′ = 2) while in BEKRES there are three. The remainder have *Z*′ = 1. The dihedral angles between the mean plane of the phenyl ring and that defined by the acetamido group as described in Section 2 vary from 1.21 (8)° in LETTIR to 28.62 (10)° in ASEDIO with most others in the 15 to 25° range.

## 5. Hirshfeld surface analysis

**Figure 5:**
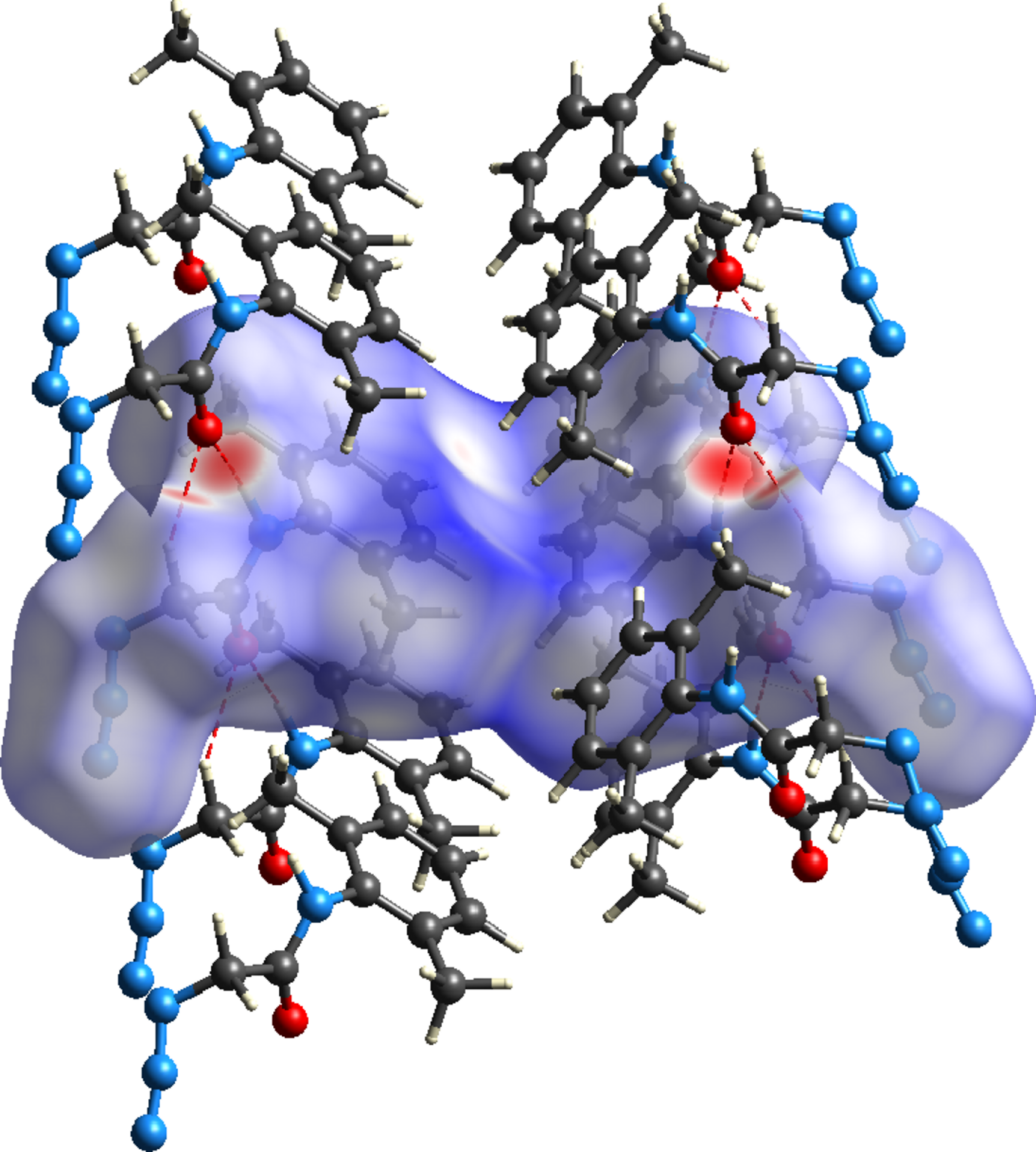
The Hirshfeld *d*_norm_ surface for the asymmetric unit with several neighboring mol­ecules. The N—H⋯O and C—H⋯O hydrogen bonds are depicted by red dashed lines.

**Figure 6:**
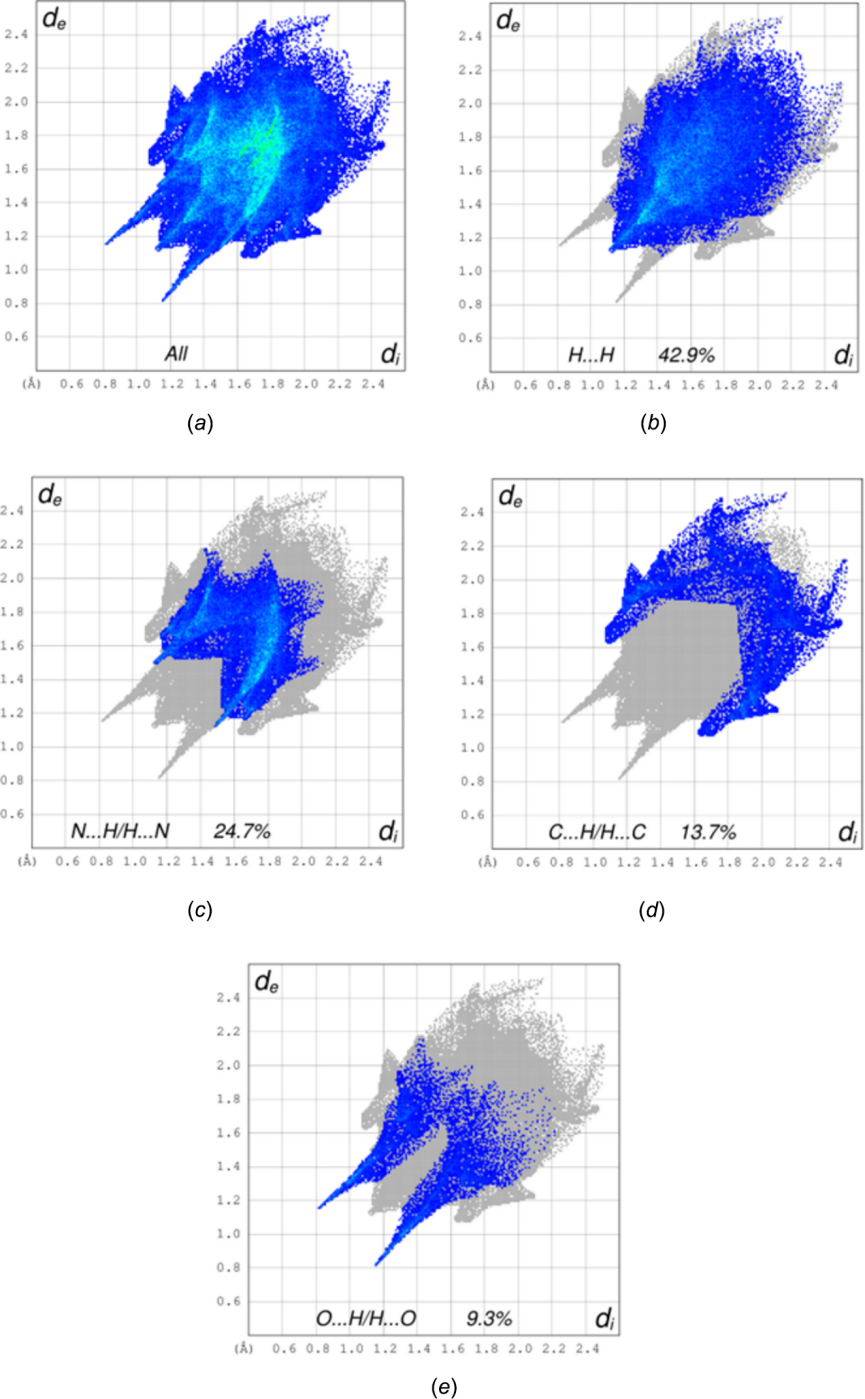
2-D fingerprint plots for all inter­molecular inter­actions (*a*) and those delineated into H⋯H (*b*), C⋯H/H⋯C (*c*), N⋯H/H⋯N (*d*) and O⋯H/H⋯O (*e*) inter­actions.

To apportion the inter­molecular inter­actions into specific atom–atom contacts, a Hishfeld surface analysis was performed with *CrystalExplorer* (Spackman *et al.*, 2021 ▸). Full descriptions of the plots obtained and their inter­pretations have been published (Tan *et al.*, 2019 ▸). Fig. 5 ▸ shows the *d*_norm_ surface together with several neighboring mol­ecules. The N—H⋯O and C—H⋯O hydrogen bonds are depicted by red dashed lines and comparison with Fig. 2 ▸ shows that this figure is another view of portions of the chain motif. The dark-red spots on the surface correspond to the N—H⋯O hydrogen bonds and the lighter red spots to the C—H⋯O hydrogen bonds. Fig. 6 ▸*a* shows the 2-D fingerprint plots for all inter­molecular contacts while Fig. 6 ▸*b*–6*e* show those delineated into H⋯H, N⋯H/H⋯N, C⋯H/H⋯C and O⋯H/H⋯O inter­actions, respectively, together with their percentage contributions. As expected, the H⋯H contacts contribute the largest amount since the hydrogen atoms constitute a large portion of the periphery of the mol­ecule. In the absence of any specific C—H⋯N hydrogen bonds, the significant contribution of N⋯H/H⋯N contacts might seem surprising, but with the azide group projecting away from the rest of the mol­ecule, there is considerable opportunity for such contacts to occur. Indeed, N2 and N6 each interact with a C—H hydrogen from a neighboring mol­ecule while the terminal nitro­gen atoms (N4 and N8) each interact with two C—H hydrogen atoms. The next largest contribution is from C⋯H/H⋯C contacts, which can be attributed to the C7—H7*C*⋯π(ring) inter­actions followed by the O⋯H/H⋯O inter­actions, which appear as a pair of sharp spikes at *d*_e_ + *d*_i_ ≃ 1.95 Å with broader shoulders at *d*_e_ + *d*_i_ ≃ 2.5 Å. These can be attributed, respectively, to the N—H⋯O and C—H⋯O hydrogen bonds. All other atom–atom contacts contribute less than 2% each, except for the N⋯N contacts which amount to 4.9%. These result from van der Waals contacts between inversion-related azide groups, which can be seen in Fig. 3 ▸.

## 6. Synthesis and crystallization

**Figure 7:**
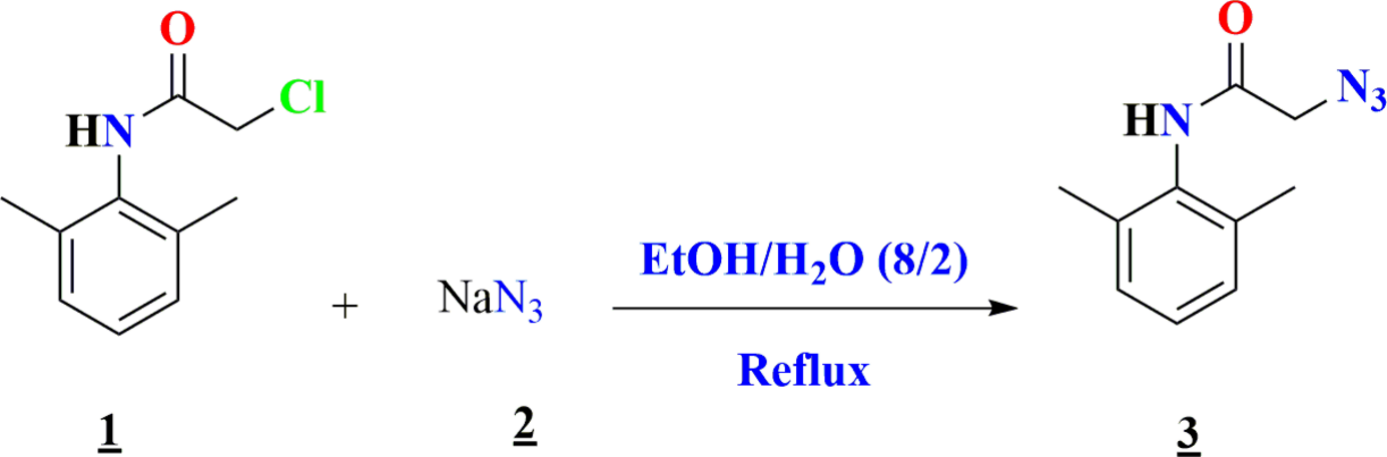
Reaction scheme for the formation of the title compound **3.**

2-Chloro-*N*-(2,6-di­methyl­phen­yl)acetamide, **1**, was obtained according to our previous work (Missioui, *et al.*, 2022*c* ▸; El Moutaouakil Ala Allah *et al.*, 2024 ▸). 2.50 mmol of compound **1** and sodium azide (3.75 mmol) were dissolved in an ethanol/water mixture (8/2) and then refluxed for 24 h at 353 K. Upon completion of the reaction (TLC), the precipitate of 2-azido-*N*-(2,6-di­methyl­phen­yl)acetamide, **3**, was filtered off and washed with cold water. The obtained precipitate was then recrystallized in ethanol. Crystals suitable for X-ray analysis were obtained by slow evaporation of the solvent (Fig. 7 ▸).

## 7. Refinement

Crystal data, data collection and structure refinement details are summarized in Table 2 ▸. Data processing revealed crystal twinning by twofold rotation around [001] and the SHELXL HKLF 5 instruction was used for refinement. In the final cycles of refinement, hydrogen-atom geometry was idealized, and a riding model was used with *U*_iso_(H) set at 1.2 or 1.5 × *U*_eq_(parent atom).

## Supplementary Material

Crystal structure: contains datablock(s) I. DOI: 10.1107/S2056989025004530/zn2043sup1.cif

Structure factors: contains datablock(s) I. DOI: 10.1107/S2056989025004530/zn2043Isup2.hkl

Supporting information file. DOI: 10.1107/S2056989025004530/zn2043Isup3.cml

CCDC reference: 2452988

Additional supporting information:  crystallographic information; 3D view; checkCIF report

## Figures and Tables

**Table 1:** Hydrogen-bond geometry (Å, °) *Cg*1 and *Cg*2 are the centroids of the C1–C6 and C11–C16 rings, respectively.

*D*—H⋯*A*	*D*—H	H⋯*A*	*D*⋯*A*	*D*—H⋯*A*
N1—H1⋯O1^i^	0.90 (8)	2.10 (8)	2.973 (6)	163 (7)
N5—H5*A*⋯O2^i^	0.91 (6)	2.13 (6)	2.995 (5)	160 (5)
C8—H8*B*⋯O1	0.96	2.47	3.046 (10)	118
C10—H10*A*⋯O1^i^	0.97	2.38	3.266 (7)	151
C18—H18*B*⋯N5	0.96	2.47	2.911 (9)	108
C20—H20*B*⋯O2^i^	0.97	2.39	3.278 (6)	152
C7—H7*C*⋯*Cg*1^i^	0.96	2.97	3.745 (7)	138
C17—H17*A*⋯*Cg*2^i^	0.96	2.87	3.722 (6)	148

Symmetry code: (i) 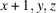.

**Table 2:** Experimental details

Crystal data	
Chemical formula	C_10_H_12_N_4_O
*M*_r_	204.24
Crystal system, space group	Triclinic, *P* 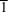
Temperature (K)	296
*a*, *b*, *c* (Å)	4.8530 (3), 7.3504 (5), 29.862 (3)
α, β, γ (°)	93.584 (6), 90.385 (5), 99.905 (5)
*V* (Å^3^)	1047.14 (13)
*Z*	4
Radiation type	Cu *K*α
μ (mm^−1^)	0.73
Crystal size (mm)	0.81 × 0.13 × 0.04

Data collection	
Diffractometer	SuperNova, Dual, Cu at home/near, Atlas
Absorption correction	Multi-scan (*CrysAlis PRO*; Rigaku OD, 2023 ▸)
*T*_min_, *T*_max_	0.245, 1.000
No. of measured, independent and observed [*I* > 2σ(*I*)] reflections	4441, 4441, 2956
*R*_int_	0.077
(sin θ/λ)_max_ (Å^−1^)	0.619

Refinement	
*R*[*F*^2^ > 2σ(*F*^2^)], *wR*(*F*^2^), *S*	0.076, 0.244, 1.08
No. of reflections	4441
No. of parameters	284
H-atom treatment	H atoms treated by a mixture of independent and constrained refinement
Δρ_max_, Δρ_min_ (e Å^−3^)	0.26, −0.28

Computer programs: *CrysAlis PRO CCD* (Rigaku OD, 2024 ▸), *CrysAlis PRO* (Rigaku OD, 2023 ▸), *SHELXT* (Sheldrick, 2015*a* ▸), *SHELXL2019/2* (Sheldrick, 2015*b* ▸), *Mercury* (Macrae *et al.*, 2020 ▸), *publCIF* (Westrip, 2010 ▸).

## supplementary crystallographic information

### 2-Azido-N-(2,6-dimethylphenyl)acetamide Crystal data

**Table:**

C_10_H_12_N_4_O	*Z* = 4
*M**_r_* = 204.24	*F*(000) = 432
Triclinic, *P*1	*D*_x_ = 1.295 Mg m^−^^3^
*a* = 4.8530 (3) Å	Cu *K*α radiation, λ = 1.54184 Å
*b* = 7.3504 (5) Å	Cell parameters from 2419 reflections
*c* = 29.862 (3) Å	θ = 5.9–72.6°
α = 93.584 (6)°	µ = 0.73 mm^−^^1^
β = 90.385 (5)°	*T* = 296 K
γ = 99.905 (5)°	Plate, yellow
*V* = 1047.14 (13) Å^3^	0.81 × 0.13 × 0.04 mm

### 2-Azido-N-(2,6-dimethylphenyl)acetamide Data collection

**Table:**

SuperNova, Dual, Cu at home/near, Atlas diffractometer	2956 reflections with *I* > 2σ(*I*)
Detector resolution: 10.5082 pixels mm^-1^	*R*_int_ = 0.077
ω scans	θ_max_ = 72.7°, θ_min_ = 4.5°
Absorption correction: multi-scan (CrysAlisPro; Rigaku OD, 2023)	*h* = −5→5
*T*_min_ = 0.245, *T*_max_ = 1.000	*k* = −8→8
4441 measured reflections	*l* = −36→34
4441 independent reflections	

### 2-Azido-N-(2,6-dimethylphenyl)acetamide Refinement

**Table:**

Refinement on *F*^2^	0 restraints
Least-squares matrix: full	Hydrogen site location: mixed
*R*[*F*^2^ > 2σ(*F*^2^)] = 0.076	H atoms treated by a mixture of independent and constrained refinement
*wR*(*F*^2^) = 0.244	*w* = 1/[σ^2^(*F*_o_^2^) + (0.1273*P*)^2^ + 0.4349*P*] where *P* = (*F*_o_^2^ + 2*F*_c_^2^)/3
*S* = 1.08	(Δ/σ)_max_ < 0.001
4441 reflections	Δρ_max_ = 0.26 e Å^−^^3^
284 parameters	Δρ_min_ = −0.28 e Å^−^^3^

### 2-Azido-N-(2,6-dimethylphenyl)acetamide Special details

**Table:**

Geometry. All esds (except the esd in the dihedral angle between two l.s. planes) are estimated using the full covariance matrix. The cell esds are taken into account individually in the estimation of esds in distances, angles and torsion angles; correlations between esds in cell parameters are only used when they are defined by crystal symmetry. An approximate (isotropic) treatment of cell esds is used for estimating esds involving l.s. planes.
Refinement. Refined as a 2-component twin.

### 2-Azido-N-(2,6-dimethylphenyl)acetamide Fractional atomic coordinates and isotropic or equivalent isotropic displacement parameters (Å^2^)

**Table:**

	*x*	*y*	*z*	*U*_iso_*/*U*_eq_	
C1	0.3914 (11)	0.7296 (8)	0.14237 (17)	0.0451 (13)	
C2	0.5308 (11)	0.9109 (9)	0.13628 (19)	0.0490 (13)	
C3	0.4650 (15)	1.0525 (10)	0.1654 (2)	0.0632 (17)	
H3	0.551146	1.173825	0.161931	0.076*	
C4	0.2775 (16)	1.0172 (10)	0.1989 (2)	0.0672 (18)	
H4	0.236213	1.113937	0.217831	0.081*	
C5	0.1491 (14)	0.8378 (11)	0.2047 (2)	0.0638 (18)	
H5	0.021724	0.814386	0.227667	0.077*	
C6	0.2078 (12)	0.6919 (9)	0.17669 (18)	0.0496 (13)	
C7	0.7373 (14)	0.9541 (11)	0.0994 (2)	0.0645 (17)	
H7A	0.806593	1.084855	0.100752	0.097*	
H7B	0.646671	0.916193	0.070856	0.097*	
H7C	0.890367	0.888951	0.103125	0.097*	
C8	0.0716 (16)	0.4984 (12)	0.1860 (3)	0.073 (2)	
H8A	0.206330	0.417190	0.182689	0.109*	
H8B	−0.081267	0.457138	0.165192	0.109*	
H8C	0.002923	0.497548	0.216077	0.109*	
C9	0.2520 (10)	0.4849 (8)	0.08358 (17)	0.0467 (13)	
C10	0.3565 (11)	0.3436 (9)	0.05193 (18)	0.0522 (14)	
H10A	0.558968	0.371779	0.050904	0.063*	
H10B	0.304729	0.221644	0.063171	0.063*	
C11	0.8588 (9)	0.6601 (6)	0.35827 (17)	0.0345 (10)	
C12	0.9938 (9)	0.8443 (7)	0.36511 (18)	0.0372 (11)	
C13	0.9229 (12)	0.9725 (8)	0.3366 (2)	0.0508 (13)	
H13	1.012905	1.094993	0.339935	0.061*	
C14	0.7215 (14)	0.9201 (9)	0.3034 (2)	0.0565 (15)	
H14	0.669115	1.008470	0.285733	0.068*	
C15	0.5969 (12)	0.7371 (9)	0.2964 (2)	0.0530 (14)	
H15	0.465969	0.703164	0.273131	0.064*	
C16	0.6628 (10)	0.6023 (7)	0.32321 (19)	0.0425 (12)	
C17	1.2093 (11)	0.9010 (8)	0.4015 (2)	0.0527 (14)	
H17A	1.372137	0.847494	0.394543	0.079*	
H17B	1.134856	0.858455	0.429465	0.079*	
H17C	1.259438	1.033412	0.404019	0.079*	
C18	0.5361 (15)	0.4039 (9)	0.3133 (3)	0.0652 (17)	
H18A	0.344075	0.384169	0.322205	0.098*	
H18B	0.636947	0.327169	0.329667	0.098*	
H18C	0.545843	0.372652	0.281742	0.098*	
C19	0.7322 (9)	0.4416 (6)	0.41601 (17)	0.0356 (10)	
C20	0.8437 (9)	0.3184 (7)	0.44798 (17)	0.0394 (11)	
H20A	0.789956	0.190184	0.436906	0.047*	
H20B	1.046425	0.347350	0.448974	0.047*	
N1	0.4497 (9)	0.5850 (7)	0.11149 (15)	0.0452 (11)	
N2	0.2389 (11)	0.3427 (8)	0.00639 (16)	0.0575 (13)	
N3	0.0103 (11)	0.2437 (8)	−0.00052 (15)	0.0552 (13)	
N4	−0.1938 (12)	0.1562 (10)	−0.0128 (2)	0.0742 (17)	
N5	0.9233 (7)	0.5280 (5)	0.38826 (15)	0.0356 (9)	
N6	0.7382 (9)	0.3418 (6)	0.49305 (17)	0.0509 (11)	
N7	0.5103 (9)	0.2455 (6)	0.50054 (15)	0.0421 (10)	
N8	0.3088 (10)	0.1657 (8)	0.5130 (2)	0.0650 (15)	
O1	0.0030 (8)	0.4970 (7)	0.08450 (15)	0.0596 (12)	
O2	0.4864 (7)	0.4538 (6)	0.41561 (14)	0.0493 (10)	
H5A	1.108 (12)	0.534 (8)	0.3946 (18)	0.040 (14)*	
H1	0.630 (16)	0.583 (11)	0.105 (2)	0.07 (2)*	

### 2-Azido-N-(2,6-dimethylphenyl)acetamide Atomic displacement parameters (Å^2^)

**Table:**

	*U*^11^	*U*^22^	*U*^33^	*U*^12^	*U*^13^	*U*^23^
C1	0.040 (3)	0.058 (4)	0.039 (3)	0.016 (2)	−0.009 (2)	−0.007 (2)
C2	0.045 (3)	0.052 (3)	0.049 (3)	0.009 (2)	−0.010 (2)	−0.003 (2)
C3	0.074 (4)	0.052 (4)	0.063 (4)	0.018 (3)	−0.009 (3)	−0.009 (3)
C4	0.086 (5)	0.068 (5)	0.051 (4)	0.026 (4)	−0.002 (3)	−0.010 (3)
C5	0.068 (4)	0.086 (5)	0.042 (3)	0.026 (4)	0.007 (3)	0.001 (3)
C6	0.051 (3)	0.057 (4)	0.042 (3)	0.013 (3)	−0.005 (2)	−0.001 (2)
C7	0.055 (4)	0.075 (5)	0.063 (4)	0.013 (3)	0.000 (3)	0.002 (3)
C8	0.076 (5)	0.081 (5)	0.059 (4)	0.004 (4)	0.008 (3)	0.016 (4)
C9	0.034 (3)	0.068 (4)	0.037 (3)	0.008 (2)	−0.0043 (19)	−0.003 (2)
C10	0.044 (3)	0.067 (4)	0.043 (3)	0.010 (3)	−0.008 (2)	−0.013 (3)
C11	0.0232 (19)	0.030 (2)	0.054 (3)	0.0115 (16)	0.0014 (18)	0.006 (2)
C12	0.029 (2)	0.028 (2)	0.057 (3)	0.0096 (17)	0.0047 (18)	0.005 (2)
C13	0.054 (3)	0.033 (3)	0.067 (4)	0.012 (2)	0.009 (3)	0.011 (2)
C14	0.072 (4)	0.048 (3)	0.055 (4)	0.024 (3)	−0.001 (3)	0.012 (3)
C15	0.056 (3)	0.056 (4)	0.051 (3)	0.021 (3)	−0.009 (2)	0.007 (3)
C16	0.037 (3)	0.038 (3)	0.053 (3)	0.011 (2)	−0.005 (2)	0.001 (2)
C17	0.037 (3)	0.048 (3)	0.070 (4)	−0.002 (2)	−0.003 (3)	0.004 (3)
C18	0.073 (4)	0.046 (4)	0.073 (4)	0.003 (3)	−0.018 (3)	−0.003 (3)
C19	0.025 (2)	0.025 (2)	0.058 (3)	0.0070 (16)	−0.0036 (18)	0.0067 (19)
C20	0.030 (2)	0.032 (2)	0.061 (3)	0.0122 (17)	0.003 (2)	0.016 (2)
N1	0.033 (2)	0.057 (3)	0.046 (2)	0.0126 (19)	−0.0036 (18)	−0.008 (2)
N2	0.046 (3)	0.080 (4)	0.043 (3)	0.004 (2)	−0.001 (2)	−0.003 (2)
N3	0.047 (3)	0.077 (4)	0.041 (3)	0.014 (2)	−0.0020 (19)	−0.008 (2)
N4	0.050 (3)	0.100 (5)	0.067 (4)	0.008 (3)	−0.016 (3)	−0.017 (3)
N5	0.0171 (16)	0.032 (2)	0.060 (3)	0.0073 (13)	−0.0003 (16)	0.0102 (17)
N6	0.044 (3)	0.044 (3)	0.062 (3)	−0.0020 (19)	−0.002 (2)	0.008 (2)
N7	0.034 (2)	0.037 (2)	0.058 (3)	0.0117 (16)	−0.0012 (17)	0.0084 (19)
N8	0.037 (3)	0.071 (4)	0.089 (4)	0.009 (2)	0.006 (2)	0.014 (3)
O1	0.031 (2)	0.085 (3)	0.062 (3)	0.0177 (19)	−0.0070 (16)	−0.016 (2)
O2	0.0211 (17)	0.058 (2)	0.073 (3)	0.0146 (15)	0.0032 (15)	0.0188 (19)

### 2-Azido-N-(2,6-dimethylphenyl)acetamide Geometric parameters (Å, º)

**Table:**

C1—C6	1.373 (8)	C12—C13	1.393 (7)
C1—C2	1.410 (9)	C12—C17	1.493 (7)
C1—N1	1.430 (7)	C13—C14	1.375 (9)
C2—C3	1.396 (8)	C13—H13	0.9300
C2—C7	1.505 (9)	C14—C15	1.379 (9)
C3—C4	1.363 (10)	C14—H14	0.9300
C3—H3	0.9300	C15—C16	1.390 (8)
C4—C5	1.380 (10)	C15—H15	0.9300
C4—H4	0.9300	C16—C18	1.492 (8)
C5—C6	1.389 (9)	C17—H17A	0.9600
C5—H5	0.9300	C17—H17B	0.9600
C6—C8	1.505 (10)	C17—H17C	0.9600
C7—H7A	0.9600	C18—H18A	0.9600
C7—H7B	0.9600	C18—H18B	0.9600
C7—H7C	0.9600	C18—H18C	0.9600
C8—H8A	0.9600	C19—O2	1.212 (5)
C8—H8B	0.9600	C19—N5	1.350 (6)
C8—H8C	0.9600	C19—C20	1.514 (6)
C9—O1	1.227 (6)	C20—N6	1.454 (7)
C9—N1	1.351 (7)	C20—H20A	0.9700
C9—C10	1.515 (7)	C20—H20B	0.9700
C10—N2	1.470 (7)	N1—H1	0.90 (8)
C10—H10A	0.9700	N2—N3	1.226 (7)
C10—H10B	0.9700	N3—N4	1.129 (7)
C11—C12	1.401 (7)	N5—H5A	0.91 (6)
C11—C16	1.405 (7)	N6—N7	1.236 (6)
C11—N5	1.433 (6)	N7—N8	1.129 (7)
			
C6—C1—C2	121.7 (6)	C11—C12—C17	121.0 (5)
C6—C1—N1	120.9 (6)	C14—C13—C12	120.8 (5)
C2—C1—N1	117.4 (5)	C14—C13—H13	119.6
C3—C2—C1	117.2 (6)	C12—C13—H13	119.6
C3—C2—C7	120.4 (6)	C13—C14—C15	120.4 (6)
C1—C2—C7	122.4 (6)	C13—C14—H14	119.8
C4—C3—C2	121.6 (7)	C15—C14—H14	119.8
C4—C3—H3	119.2	C14—C15—C16	121.4 (5)
C2—C3—H3	119.2	C14—C15—H15	119.3
C3—C4—C5	119.9 (7)	C16—C15—H15	119.3
C3—C4—H4	120.0	C15—C16—C11	117.4 (5)
C5—C4—H4	120.0	C15—C16—C18	120.3 (5)
C4—C5—C6	120.7 (6)	C11—C16—C18	122.3 (5)
C4—C5—H5	119.6	C12—C17—H17A	109.5
C6—C5—H5	119.6	C12—C17—H17B	109.5
C1—C6—C5	118.8 (6)	H17A—C17—H17B	109.5
C1—C6—C8	122.9 (6)	C12—C17—H17C	109.5
C5—C6—C8	118.3 (6)	H17A—C17—H17C	109.5
C2—C7—H7A	109.5	H17B—C17—H17C	109.5
C2—C7—H7B	109.5	C16—C18—H18A	109.5
H7A—C7—H7B	109.5	C16—C18—H18B	109.5
C2—C7—H7C	109.5	H18A—C18—H18B	109.5
H7A—C7—H7C	109.5	C16—C18—H18C	109.5
H7B—C7—H7C	109.5	H18A—C18—H18C	109.5
C6—C8—H8A	109.5	H18B—C18—H18C	109.5
C6—C8—H8B	109.5	O2—C19—N5	124.4 (4)
H8A—C8—H8B	109.5	O2—C19—C20	120.5 (4)
C6—C8—H8C	109.5	N5—C19—C20	115.1 (4)
H8A—C8—H8C	109.5	N6—C20—C19	111.9 (4)
H8B—C8—H8C	109.5	N6—C20—H20A	109.2
O1—C9—N1	124.2 (5)	C19—C20—H20A	109.2
O1—C9—C10	120.9 (5)	N6—C20—H20B	109.2
N1—C9—C10	114.8 (4)	C19—C20—H20B	109.2
N2—C10—C9	111.5 (5)	H20A—C20—H20B	107.9
N2—C10—H10A	109.3	C9—N1—C1	122.5 (4)
C9—C10—H10A	109.3	C9—N1—H1	118 (5)
N2—C10—H10B	109.3	C1—N1—H1	117 (5)
C9—C10—H10B	109.3	N3—N2—C10	115.4 (5)
H10A—C10—H10B	108.0	N4—N3—N2	170.7 (6)
C12—C11—C16	122.0 (5)	C19—N5—C11	122.4 (3)
C12—C11—N5	118.5 (4)	C19—N5—H5A	119 (4)
C16—C11—N5	119.5 (4)	C11—N5—H5A	115 (4)
C13—C12—C11	117.9 (5)	N7—N6—C20	115.9 (5)
C13—C12—C17	121.1 (5)	N8—N7—N6	171.1 (6)

### 2-Azido-N-(2,6-dimethylphenyl)acetamide Hydrogen-bond geometry (Å, º)

*Cg*1 and *Cg*2 are the centroids of the C1–C6 and C11–C16 rings, respectively.

**Table:**

*D*—H···*A*	*D*—H	H···*A*	*D*···*A*	*D*—H···*A*
N1—H1···O1^i^	0.90 (8)	2.10 (8)	2.973 (6)	163 (7)
N5—H5*A*···O2^i^	0.91 (6)	2.13 (6)	2.995 (5)	160 (5)
C8—H8*B*···O1	0.96	2.47	3.046 (10)	118
C10—H10*A*···O1^i^	0.97	2.38	3.266 (7)	151
C18—H18*B*···N5	0.96	2.47	2.911 (9)	108
C20—H20*B*···O2^i^	0.97	2.39	3.278 (6)	152
C7—H7*C*···*Cg*1^i^	0.96	2.97	3.745 (7)	138
C17—H17*A*···*Cg*2^i^	0.96	2.87	3.722 (6)	148

Symmetry code: (i) *x*+1, *y*, *z*.
